# Toroidal Dipole‐Dominated High‐Performance Optical Sensors via Nanoimprinted Tetramer Architectures

**DOI:** 10.1002/nap2.70336

**Published:** 2026-10-21

**Authors:** Hongsen Zhao, Shuai Han, Youpeng Zhuo, Ang Xu, Zhixin Wan, Yimin Chen, Peiqing Zhang, Qiushun Zou, Xiang Shen

**Affiliations:** ^1^ Faculty of Electrical Engineering and Computer Science Ningbo University Ningbo China; ^2^ Zhejiang Key Laboratory of Advanced Optical Functional Materials and Devices Engineering Research Center for Advanced Infrared Photoelectric Materials and Devices of Zhejiang Province Ningbo University Ningbo China; ^3^ Key Laboratory of Green Fabrication and Surface Technology of Advanced Metal Materials Ministry of Education School of Materials Science and Engineering Anhui University of Technology Ma'anshan China

**Keywords:** nanoimprint, optical sensor, tetramer, toroidal dipole

## Abstract

Practical high‐Q metasurface sensors face two bottlenecks: maximizing analyte near‐field coupling while suppressing radiative loss and ensuring compatibility with low‐cost large‐area fabrication. Here, we present a nanoimprinted tetramer architecture (NTA) sensor fabricated via two‐step nanoimprint lithography, consisting of polystyrene nanopillar tetramer arrays atop a PS waveguide and gold mirror, supporting a prominent resonance mode at 810 nm (D_1_ mode) exhibiting a pronounced Fano line shape with a high *Q* of 3124. In contrast, the experimentally measured resonance of the fabricated device exhibits a FWHM of approximately 12 nm, corresponding to a *Q* factor of about 70. Multipole decomposition confirms that the D_1_ mode is governed by toroidal dipoles, with suppressed conventional electric/magnetic multipoles, thereby achieving radiative cancellation via multipole manipulation. Measured in aqueous glycerol solutions, the sensor achieves a refractive index sensitivity of 501 nm/RIU and a figure of merit of 42 RIU^−1^. Label‐free hemoglobin biosensing yields a sensitivity of 354.7 nm·(μg/L)^−1^. Benefiting from tetramer symmetry, the resonance response remains stable across 0°–90° polarization angles. This work develops a scalable low‐cost strategy for toroidal‐dipole‐enhanced high‐Q biosensors, promising high‐sensitivity detection for medical diagnosis and environmental monitoring.

## Graphical abstract

We developed a scalable low‐cost nanoimprinted tetramer architecture (NTA) sensor using two‐step nanoimprint lithography. It comprises polystyrene nanopillar tetramer arrays on a PS waveguide and gold mirror, supporting a toroidal‐dipole‐mediated resonant mode at 810 nm with a simulated Q of 3124. It achieves a refractive‐index sensitivity of 501 nm/RIU and label‐free hemoglobin detection at 354.7 nm·(μg/L)⁻¹, with a stable response over 0‐90° polarization, providing a manufacturable platform for high‐performance toroidal‐dipole‐enhanced biosensing.

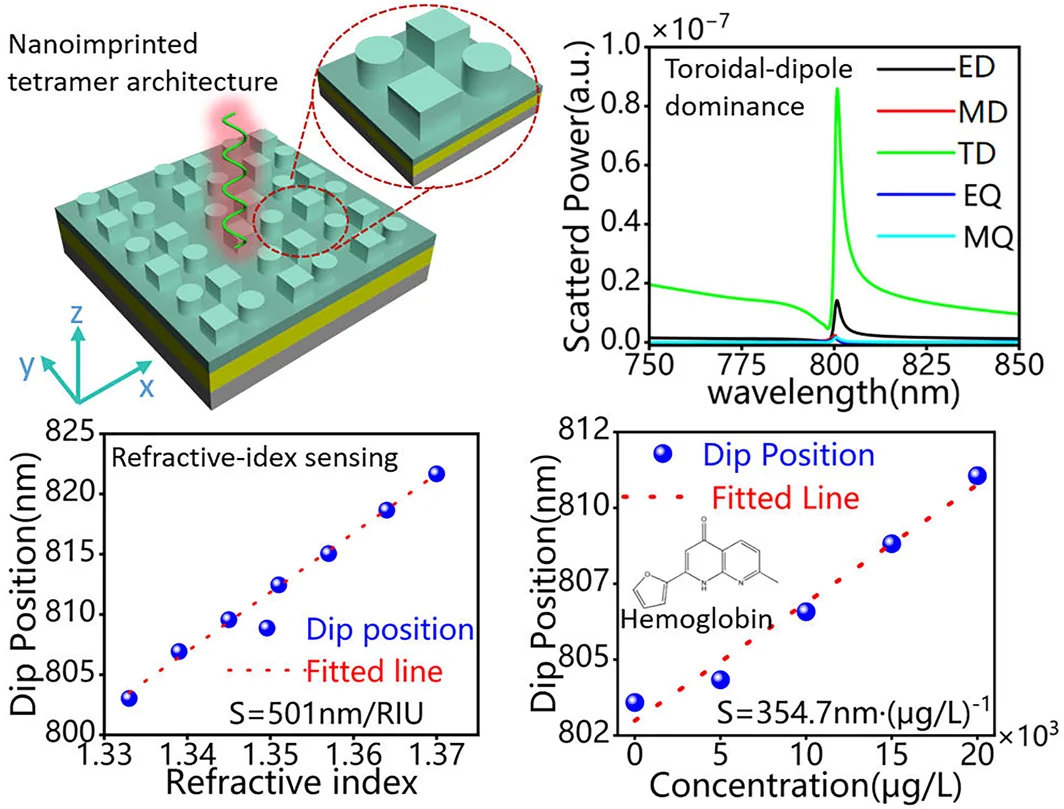

## 1 Introduction

Optical sensors have emerged as indispensable devices in biosensing, environmental monitoring, and medical diagnosis because of their high sensitivity, low detection limits, nondestructive testing, and real‐time response [1, 2, 3]. Recently, with the rapid development of nanophotonics, resonant periodic nanostructures (e.g., gratings and nanopyramids) have been widely integrated into optical sensor design [4, 5, 6, 7]. These periodic structures can concentrate electromagnetic energy into subwavelength volumes, thereby amplifying light–matter interactions and endowing sensors with ultra‐high sensing performances, laying a solid foundation for the development of ultra‐compact high‐precision optical sensing devices [8]. A key performance objective in optical sensors is to generate spectral features that are simultaneously highly sensitive to refractive‐index perturbations and sufficiently narrow to satisfy high detection resolution. In practice, this requires suppressing radiative leakage and parasitic loss channels while maintaining strong evanescent‐field overlap with the analyte.

From a manufacturing perspective, electron beam lithography (EBL) has become the mainstream approach for fabricating metasurfaces due to its high resolution and design flexibility, but it is intrinsically limited by high cost and low throughput, which restricts scalable applications [9]. Nanoimprint lithography (NIL) offers a compelling alternative, whereas EBL may still be used to fabricate the initial master template [10, 11, 12, 13, 14, 15]. NIL enables rapid replication of nanoscale patterns with reusable elastomer stamps, dramatically reducing per‐device cost and enabling large‐scale production [16, 17]. Consequently, NIL is increasingly recognized as a viable manufacturing route for functional nanophotonic devices, particularly when combined with replication strategies that preserve fidelity across repeated imprints.

Recently, extensive exploration of multipolar excitations beyond the conventional electric dipole (ED) and magnetic dipole (MD) has been motivated by enhanced high‐Q resonances and near‐field confinement [18, 19, 20]. The toroidal dipole (TD) is a distinct electromagnetic excitation arising from poloidal currents, fundamentally different from electric and magnetic dipoles. Unlike anapole states that rely on precise ED‐TD interference, a pure TD‐dominated resonance achieves far‐field radiation suppression with strong near‐field confinement without delicate multipolar cancellation [21, 22, 23, 24, 25]. This makes TD resonances attractive for high‐Q sensing: suppressed radiative loss yields ultranarrow linewidths, while confined near fields maximize analyte overlap [26, 27, 28, 29, 30]. Although toroidal resonances have been observed in metallic and dielectric metasurfaces, many demonstrations remain polarization‐sensitive and rely on fabrication approaches that are not readily scalable, leaving a gap between fundamental toroidal physics and practical sensing platforms [31, 32, 33, 34, 35].

In this work, we proposed and experimentally demonstrated a novel nanoimprinted tetramer architecture (NTA) sensor, comprising PS tetramer nanopillars integrated with a waveguide layer and a gold film on a silicon substrate. The tetramer architecture is designed to enhance light–matter interaction through near‐field coupling and symmetry‐enabled multipolar control, producing two resonance modes: D_1_, at 810 nm, dominated by toroidal dipoles with a high *Q* (= 3124) and strong localized field enhancement, and D_2_, at 940 nm. The device is fabricated using a two‐step NIL strategy that leverages the reusability of the silicon master template and polydimethylsiloxane (PDMS) subtemplate, enabling high‐efficiency low‐cost production. Experimentally, the device maintains a stable optical response across polarization angles from 0° to 90°, and it demonstrates strong sensing performance with refractive index sensitivity of 501 nm/RIU and biosensing sensitivities of 127.5 nm·(μg/L)^−1^ (creatinine) and 354.7 nm·(μg/L)^−1^ (hemoglobin). Collectively, the results indicate that the primary performance gains stem from toroidal‐dominant multipolar engineering and radiation suppression, offering a manufacturable route to high‐Q nanophotonic biosensing.

## 2 Results and Discussion

The geometry of the proposed NTA sensor and its unit cell are shown in Figure 1a. The NTA consists of a silicon substrate, a gold film, a PS waveguide layer, and an array of periodic PS tetramer nanopillars, where these nanopillars are fabricated directly on the waveguide layer via nanoimprint lithography. The PS tetramer arrangement is chosen for its manufacturability and its ability to support strong near‐field coupling and high surface sensitivity, relative to more complex meta‐atom geometries [36, 37, 38, 39]. To systematically tune the resonance behavior, the unit cell is parameterized by the side length of square nanopillars (*l* = 400 nm), the radius of circular nanopillars (*r* = 200 nm), the height of these nanopillars (*h* = 300 nm), the gap between the nanopillars (*x* = *y* = 200 nm), and the period (*p* = 1.2 μm). Using the finite‐difference time‐domain (FDTD) method, the reflection spectrum is calculated under normal incidence along the *z* direction, with the electric field polarized along the *x* axis and the magnetic field polarized along the *y* axis (Figure 1b), revealing two resonance dips: one at 810 nm (D_1_ mode) and another at 940 nm (D_2_ mode). Specifically, we used a refractive index of 1.5717 for PS [40], applied the Johnson–Christy dispersion model for gold [41], and used silicon refractive‐index data from Ref. [42]. The D_1_ mode exhibits a notably narrow linewidth (FWHM = 0.26 nm), yielding a high *Q* (=3124), and the spectral profile shows an asymmetric Fano line shape, indicating interference between a narrow discrete resonance and a broad background scattering channel.

**FIGURE 1:**
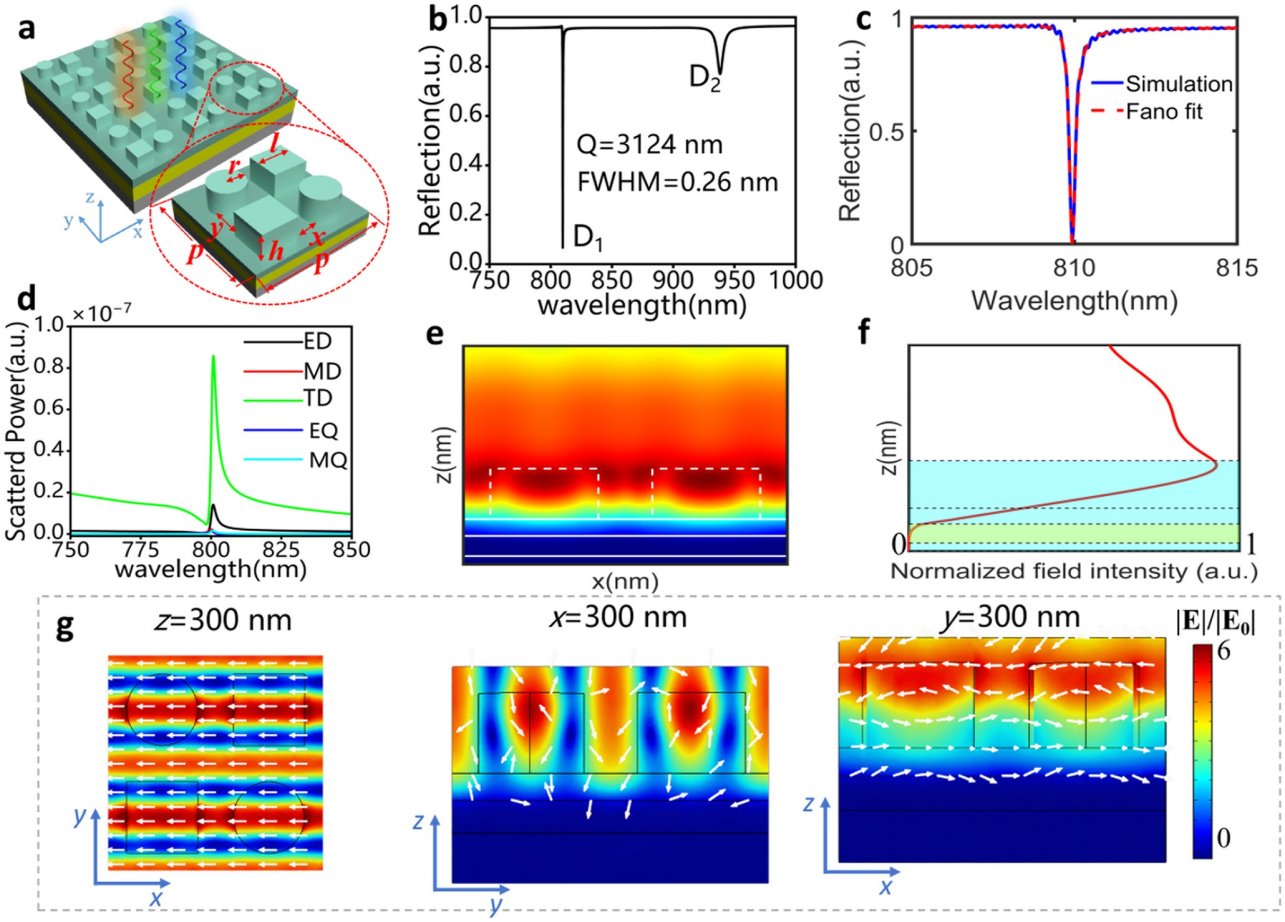
Geometry and optical properties of the nanoimprinted tetramer architecture (NTA) sensor. (a) Schematic diagram of the NTA and its unit cell (*l* = 400 nm, *r* = 200 nm, *h* = 300 nm, *x* = *y* = 200 nm, and *p* = 1.2 μm). (b) Simulated spectrum of the NTA sensor, where two resonant dips (D_1_, D_2_) are separately labeled. D_1_ mode exhibits a Q‐factor of 3124 and FWHM of 0.26 nm. (c) Fano fitting of the D_1_ mode (red dashed line), consistent with the simulated spectrum (blue solid line). (d) Cartesian multipole decomposition of the D_1_ mode. EQ: Electric quadrupole and MQ: magnetic quadrupole. (e) The total electric field enhancement distribution and (f) electric field intensity map for the D_1_ mode. (g) The electric field enhancement distributions of the D_1_ mode at the cross‐sections of *z* = 300 nm, *x* = 300 nm, and *y* = 300 nm.

As shown in Figure 1c, the D_1_ mode spectrum is well fitted by a typical Fano formula [43, 44]:

(1) $$\begin{array}{c} R={\left|a_{1}+ja_{2}+\frac{b}{\omega -\omega_{0}+j\gamma }\right|}^{2} \end{array},$$

where *ω* is the resonant frequency and *a*_*1*_ *, a*_*2,*_ and *b* are constant real numbers, whose values are 0.0697, −0.976, and −0.1119, respectively. Moreover, *ω* is the swept frequency, *ω*_*0*_ the resonant frequency, and *γ* the damping rate. The close agreement between the fitted and simulated spectra verifies the asymmetric Fano line shape of the D_1_ mode. The D_2_ resonance can likewise be described by a Fano profile as shown in Supporting Information S1: Figure S1.

To elucidate the physical origin of the D_1_ mode, we performed Cartesian multipole decomposition using the finite‐element method (FEM). The wavelength‐dependent scattered powers of the ED, MD, TD, EQ, and MQ contributions are shown in Figure 1d. The TD contribution shows a pronounced maximum and remains nearly one order of magnitude larger than the conventional multipolar contributions at 810 nm, meaning that the D_1_ mode is dominant. This TD‐dominated response is consistent with reduced radiative leakage and the ultranarrow linewidth of D_1_ mode. For qualitative comparison, wavelength‐dependent multipole scattering powers from FEM calculations were summed, and the resulting total multipole spectral response was compared with the reflectance spectrum computed independently using FDTD. Multipole data obtained from FEM were not incorporated into the FDTD model. The minor spectral discrepancy between the two results mainly originates from the inherent differences between the FEM and FDTD methods.

For this rotationally symmetric structure, rotating the incident polarization maps the response onto a symmetry‐equivalent orthogonal channel, thereby preserving the stable D_1_ resonance. This mechanism is consistent with recent reports of polarization‐insensitive resonances via structural symmetry engineering. To further elucidate the physical origin of the D_1_ mode, the cross‐sectional electric‐field vector distributions are presented in Supporting Information S1: Figure S2. As shown in the two *x*–*z* planes passing through the constituent nanopillars, pronounced local circulating electric‐field patterns are observed within the individual resonators. Such circulating fields are associated with localized magnetic responses in dielectric resonators and provide a near‐field signature of the collective toroidal excitation. Therefore, the field distributions in Figure 1g and Supporting Information S1: Figure S2 are consistent with the multipole decomposition, which identifies the D_1_ mode as being dominated by the toroidal dipole contribution.

In contrast, the D_2_‐mode decomposition indicates an MD‐dominated response across the entire wavelength range, with a strong *y*‐component contribution, suggesting a distinct physical mechanism for the D_2_ mode (Supporting Information S1: Figure S3a,b). The strong suppression of conventional dipolar channels in the D_1_ mode, accompanied by a dominant toroidal response, indicates that the D_1_ mode arises from multipolar interference and radiation cancellation engineered by symmetric tetramer coupling. The D_2_ mode is an MD‐dominated hybrid resonance resulting from coupling between the PS waveguide and surface plasmons at a gold interface, not from multipolar interference. This gives it a broader linewidth, lower *Q*, and weaker environmental response than the D_1_ mode. The scattering spectra show that the TD resonance is *x*‐component‐dominant and the MD resonance is *y*‐component‐dominant, consistent with the incident E‐field along *x* and H‐field along the *y*‐axis. The tetramer's rotational symmetry suppresses orthogonal contributions, accounting for the observed polarization insensitivity.

Near‐field distributions provide further mechanistic insight into why the D_1_ mode yields excellent sensing performance. The total electric field distribution and field intensity map (Figure 1e,f) show that, for the D_1_ mode, the electric field is strongly concentrated at the surface and gap regions of the PS nanopillar tetramer (Figure 1g), achieving a pronounced localized field enhancement. The white arrows in Figure 1g indicate the direction of the electric field. The optical mode effectively overlaps with the surrounding medium above the metasurface. This field localization is particularly favorable for refractive‐index and biosensing applications as it maximizes the interaction volume between the evanescent field and analytes near the nanopillar surfaces and in the inter‐nanopillar gaps. Moreover, we constructed a free‐space reference model containing only the tetramer by eliminating a gold mirror, silicon substrate, and PS waveguide. Its multipole decomposition (Supporting Information S1: Figure S4) shows that the toroidal dipole (TD) dominates near the D_1_ resonance, consistent with the results for the full structure. Although the substrate, mirror, and waveguide alter the local electromagnetic environment and the relative multipole amplitudes, they do not change the dominant multipole character of D_1_. Hence, the D_1_ resonance originates from a toroidal‐dipole‐dominated mode. By contrast, the D_2_ mode shows field confinement near the PS waveguide layer and the gold film interface (Supporting Information S1: Figure S5), consistent with exciting surface plasmons at the gold‐PS interface. This implies reduced environmental sensitivity for the D_2_ mode because the dominant field energy is bound closer to the metal–dielectric interface and less exposed to the external analyte medium. Although the electric‐field vectors in the *x*–*y* cross section exhibit an apparently electric‐dipole‐like pattern, the two *x*–*z* cross sections reveal local electric‐field circulation within the nanopillars, indicating the near‐field character of a magnetic‐dipole response (Supporting Information S1: Figure S5e). This feature is consistent with the dominant MD scattering contribution at D_2_ in Supporting Information S1: Figure S3a, supporting the identification of D_2_ as an MD‐dominated resonance. Therefore, we select the D_1_ mode as the primary sensing resonance owing to its advantageous combination of well‐resolved spectral features, strong field overlap with the analyte, and well‐defined multipolar origin. As shown in Figure 1g, the D_1_ electromagnetic field is concentrated primarily at nanopillar surfaces and interpillar gaps, facilitating enhanced interaction with the surrounding sensing medium.

A systematic optimization study is conducted to identify the geometric parameter set that maximizes D_1_ mode performance. When the side length *l* of square nanopillars increases from 300 to 500 nm (with *r* = 200 nm, *h* = 300 nm, *x* = *y* = 200 nm, and *p* = 1.2 μm), the D_1_ mode undergoes a redshift, and the resonance absorption intensity increases slightly (Figure 2a). The FWHM increases approximately linearly with length *l*, corresponding to a monotonic reduction in *Q* (Figure 2b), indicating that larger feature sizes increase radiative coupling or broaden the resonance by altering the multipole balance. Similar behavior is observed when varying the radius *r* of the circular nanopillars from 150 to 250 nm; the resonance redshifts, the FWHM increases linearly, and *Q* decreases (Figure 2c,d). The resonance intensity reaches a maximum around *l* = 400 nm and *r* = 200 nm, which are chosen to balance strong resonance contrast with minimal linewidth. In addition, we plotted the full dispersion curves for varying *l* and *r* (Supporting Information S1: Figure S6). The shifts of modes D_1_ and D_2_ are marked with white dashed lines.

**FIGURE 2:**
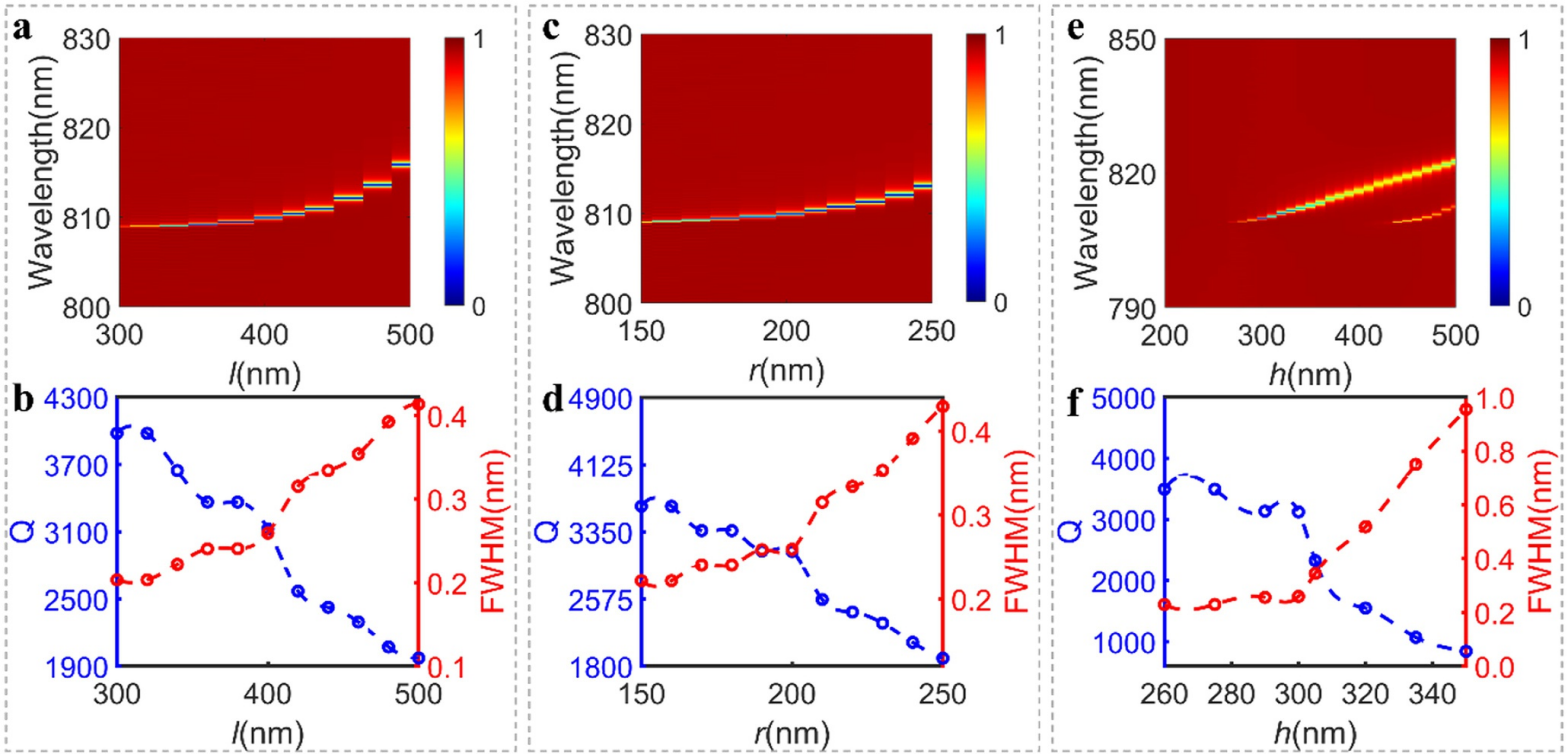
Optimal geometric parameters of the NTA. (a) The reflection map of the NTA with the side length *l* of square nanopillars varying from 300 to 500 nm. (b) The FWHM and Q of the D_1_ mode with the side length *l*. (c) The reflection map of the NTA with the radius *r* of circular nanopillars from 150 to 250 nm. (d) The FWHM and Q of the D_1_ mode as functions of the radius *r*. (e) The height‐modulated reflection map of the NTA with the height h from 200 to 500 nm. (f) The FWHM and Q of the D_1_ mode as functions of the height *h*.

Meanwhile, we performed parametric sweeps to examine how geometric parameters modulate the D_1_ and D_2_ resonant properties. As *l* and *r* are varied, the *Q* factor decreases gradually with a concomitant increase in FWHM; no prominent Q‐factor enhancement or vanishing linewidth emerges at any specific parameter. Moreover, both D_1_ and D_2_ evolve continuously with *l* and *r* without mode termination, linewidth collapse, or loss of far‐field resonance. For square and circular nanopillars with equal cross‐sectional areas, the side length of the square is *l* ˜ 354.5 nm for *r* = 200 nm, whereas the radius of the circular pillar becomes *r* ˜ 225.7 nm when *l* = 400 nm. Nevertheless, no distinct Q‐factor peak or divergence appears around these equal‐area points. These parametric results do not exhibit the typical quasi‐BIC signatures associated with equal‐area conditions or geometric asymmetry. Accordingly, our parameter sweeps provide no clear evidence that the D_1_ and D_2_ resonances stem from BIC‐to‐quasi‐BIC transitions (Supporting Information S1: Figure S6). Among these structural parameters, the nanopillars' height *h* plays a critical role in mode coupling. The height‐modulated reflection maps are shown in Figure 2e. It was observed that the D_1_ mode appears only for *h* ≥ 260 nm, suggesting a threshold for establishing the vertical confinement and coupling required for toroidal‐dominant interference. As *h* increases to 400 nm, a new resonance (D3) appears near 800 nm with different multipole character and a distinct field distribution (Supporting Information S1: Figure S7), and the *Q* of the D_1_ mode drops rapidly once *h* exceeds 300 nm. In contrast, the FWHM increases, indicating that excessive height introduces additional competing resonances and degrades mode purity. Furthermore, the vector‐field distributions of D_3_ provide additional insight into its local magnetic response. As shown in Supporting Information S1: Figure S7e, the electric field in the *y*–*z* plane exhibits local circulation‐like patterns within the nanopillars, whereas the magnetic‐field distribution in the *x*–*z* plane shows localization inside the pillars. These complementary field distributions qualitatively illustrate the local magnetic character of this mode. The multipole decomposition further shows that the magnetic quadrupole (MQ) makes the largest contribution at the D_3_ resonance, with a significant contribution from the toroidal dipole (TD) (Supporting Information S1: Figure S7b). Accordingly, D_3_ can be described as an MQ‐dominated mixed multipolar response with appreciable TD participation, whose near‐field distribution reflects the combined contributions of multiple multipolar components. Additional sweeps of the spacing *x* and *y* exhibit clear symmetry in their influence, with *Q* maximized at *x* = *y* = 200 nm (Supporting Information S1: Figure S8a–d), consistent with the tetramer symmetric coupling design. The period *p* of an NTA also affects resonance intensity as shown in Supporting Information S1: Figure S8e,f of the supporting information. As the *p* exceeds 1.2 μm, the resonant intensity decreases significantly, and changes in *Q* and FWHM of the D_1_ mode become less favorable. Collectively, the optimized configuration is determined to be *l* = 400 nm, *r* = 200 nm, *h* = 300 nm, *x* = *y* = 200 nm, and *p* = 1.2 μm, which yields the reported high *Q* and strong near‐field enhancement for the D_1_ mode.

To translate this design into a manufacturable device, we implement a two‐step NIL fabrication strategy. In step I, a silicon master template is fabricated by spin‐coating electronic resist on silicon, patterning by electron‐beam lithography (EBL), transferring the nanopillar features via inductively coupled plasma (ICP) etching, and removing residual electronic resist. Here, EBL was only used to fabricate the silicon master, from which a hard‐PDMS subtemplate was replicated to imprint the tetramer nanostructures into the PS layer. EBL relies on serial point‐by‐point exposure, whereas NIL transfers patterns from a prefabricated template and is well suited for high‐throughput, large‐area fabrication. This eliminates the need for repeated high‐resolution direct lithography for each sample as the master and subtemplate can be reused across multiple cycles. The approach lowers device cost, improves production throughput, and enables scalable large‐area batch manufacturing with faithful nanostructure replication. Hard polydimethylsiloxane (PDMS) is then cast on the silicon master, cured, and peeled off to create a reusable PDMS subtemplate. In step 2, a 120 nm‐thick gold film is deposited on a silicon substrate, a PS layer is formed on the gold film by spin‐coating, and the hard PDMS subtemplate is conformally pressed onto the PS layer at 140°C under uniform pressure from a 1.5 kg weight. After a 15 min imprinting step and a 40 min pressure‐holding period, the sample is cooled to 40°C before demolding, fully transferring the tetramer nanopillar patterns and yielding the final NTA sensors. SEM images (Figure 3b) confirm the successful replication of the tetramer nanopillar array.

**FIGURE 3:**
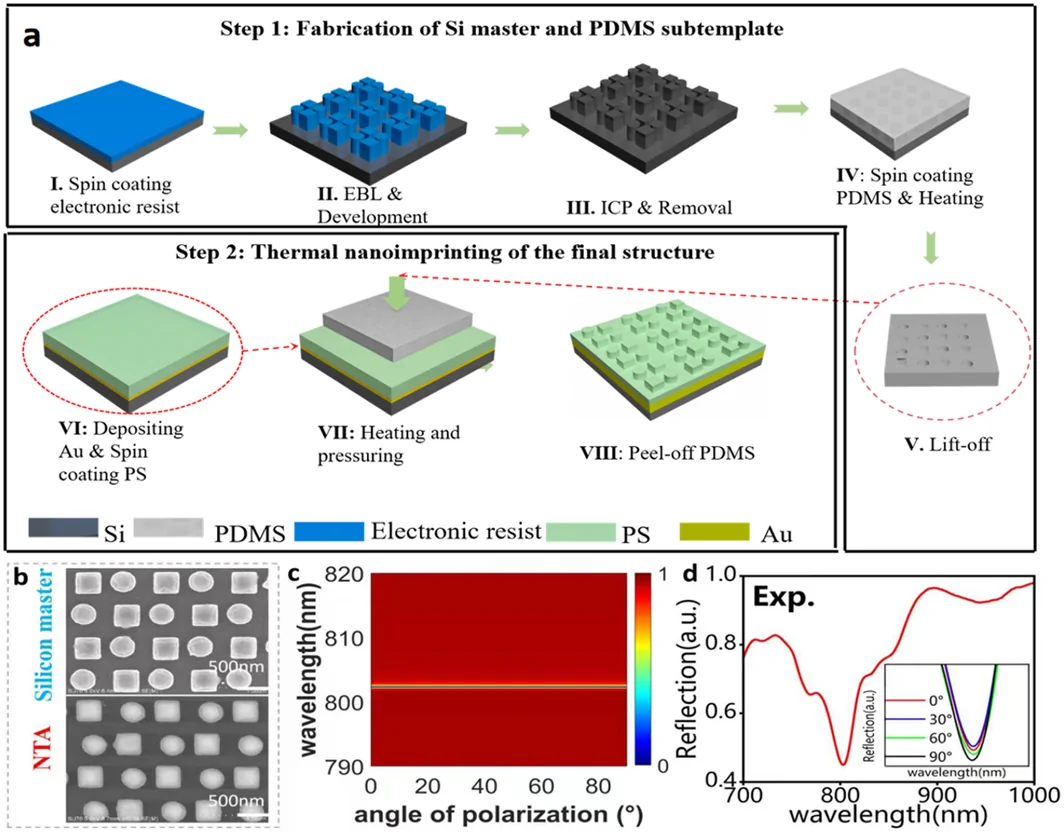
The fabrication process and polarization‐insensitive properties of an NTA. (a) A schematic of the fabrication procedure of NTA. (b) Scanning electron microscope (SEM) images of the silicon template and NTA. (c) The reflection map of the NTA with the angles of polarization varying from 0° to 90°. (d) The experimental spectra of NTA at angles of polarization of 0°, 30°, 60°, and 90°.

The slight structural deviations observed between the silicon master template and the fabricated NTA can be attributed to differences in the thermal expansion coefficients of PS and PDMS during thermal nanoimprinting. The corresponding CCD images are presented in Supporting Information S1: Figure S9a,b of the Supporting Information. The designed nanostructure has a unit‐cell period of 1.2 μm, and the rectangular array extends to more than 80 unit cells along one dimension, as verified by these CCD micrographs. Nevertheless, full‐scale finite‐array simulations with numerous unit cells are computationally intractable given our resource constraints. As reported in prior work, plasmonic grating structures can exhibit complete target functionalities with as few as 7 periods [45]. Our experimental array possesses over 80 unit cells along one dimension (Supporting Information S1: Figure S9a,b), far exceeding the benchmark value. Although finite‐array truncation still breaks translational symmetry and induces edge scattering that contributes to simulation‐experiment deviation, a sufficiently large array justifies using periodic boundary conditions in our FDTD simulations.

The experimentally measured resonance has a broader linewidth than predicted by the ideal infinite‐periodic FDTD model. Such broadening arises from a combination of finite‐array truncation, fabrication inhomogeneity, surface roughness, intrinsic material loss, and instrumental resolution. We further assessed how gold loss affects the resonant mode properties. In FDTD simulations, the gold permittivity was assigned three sets of values: Re*(ε)* and Im*(ε)*, *0.9*Re*(ε)* and *2*Im*(ε)*, and *0.8*Re*(ε)* and *3*Im*(ε)*. The corresponding resonance spectra were computed, and the *Q* factor and FWHM were quantitatively extracted using MATLAB (Supporting Information S1: Figure S10). As Re*(ε)* decreases and Im*(ε)* increases, the resonance FWHM gradually rises whereas the *Q* factor drops. This confirms that elevated metallic loss broadens the resonance and degrades the mode *Q* factor. Taken together with the above simulation results, these observations indicate that the experimentally observed linewidth broadening relative to the ideal infinite‐periodic FDTD model arises not from a single dominant source but from multiple nonidealities, such as surface roughness and realistic material losses. The polarization‐stable response of the NTA tetramer primarily arises from its in‐plane symmetry. This symmetry yields nearly identical electromagnetic coupling environments along the two orthogonal in‐plane directions, so different linearly polarized light excites resonances under comparable conditions. As the incident polarization angle varies, only the relative excitation strength of the two orthogonal channels varies, whereas the resonance wavelength and linewidth remain nearly constant [46, 47, 48, 49]. Further analysis of the toroidal‐dipole components shows that the response near the D_1_ mode is dominated by the Tx component under the investigated polarization, with much weaker Ty and Tz contributions (Supporting Information S1: Figure S11), consistent with the incident polarization used in the simulations. The polarization response of an NTA is examined both numerically and experimentally. In simulations, we calculate reflection spectra for polarization angles from 0° to 90° (defined as the angle between the incident electric field and the *x*‐axis). For the D_1_ mode, there is no appreciable change in resonance wavelength, intensity, or linewidth across the polarization range, indicating that the resonance is insensitive to polarization angle. Experimentally measured spectra at representative polarization angles (0°, 30°, 60°, 90°) show a fixed resonance wavelength for D_1_ mode with only minor intensity fluctuations consistent with simulation. This polarization robustness directly follows from the symmetric tetramer architecture and isotropic coupling pathways in the unit cell. Importantly, this feature is best positioned not as the sole innovation but as a practical advantage that simplifies operation (no polarization alignment) and improves anti‐interference capability in real sensing environments.

Refractive‐index sensing performance is first evaluated in simulation by varying the environmental refractive index and tracking the resonance shift of the D_1_ mode. As shown in Figure 4a, the resonance wavelength shows approximately linear dependence on refractive index, yielding a simulated bulk sensitivity *S* of 568 nm/RIU. Accordingly, the FOM, defined to incorporate both the bulk sensitivity *S* and the resonance linewidth (FOM = *S*/FWHM, reflecting the overall resolving power), reaches up to 2192 RIU^−1^, highlighting the benefit of simultaneously achieving high sensitivity and ultranarrow FWHM by toroidal‐dominant high‐Q resonance. Moreover, surface sensitivity is evaluated by coating the structure with a 10‐nm‐thick layer with a refractive index of 1.5 as an effective biomolecular layer; the resulting spectral shift yields a reported surface sensitivity of 2.1 nm/nm. PS exhibits good biocompatibility, chemical stability, and ease of surface modification, making it a promising substrate for surface functionalization in biomolecular sensing [50].

**FIGURE 4:**
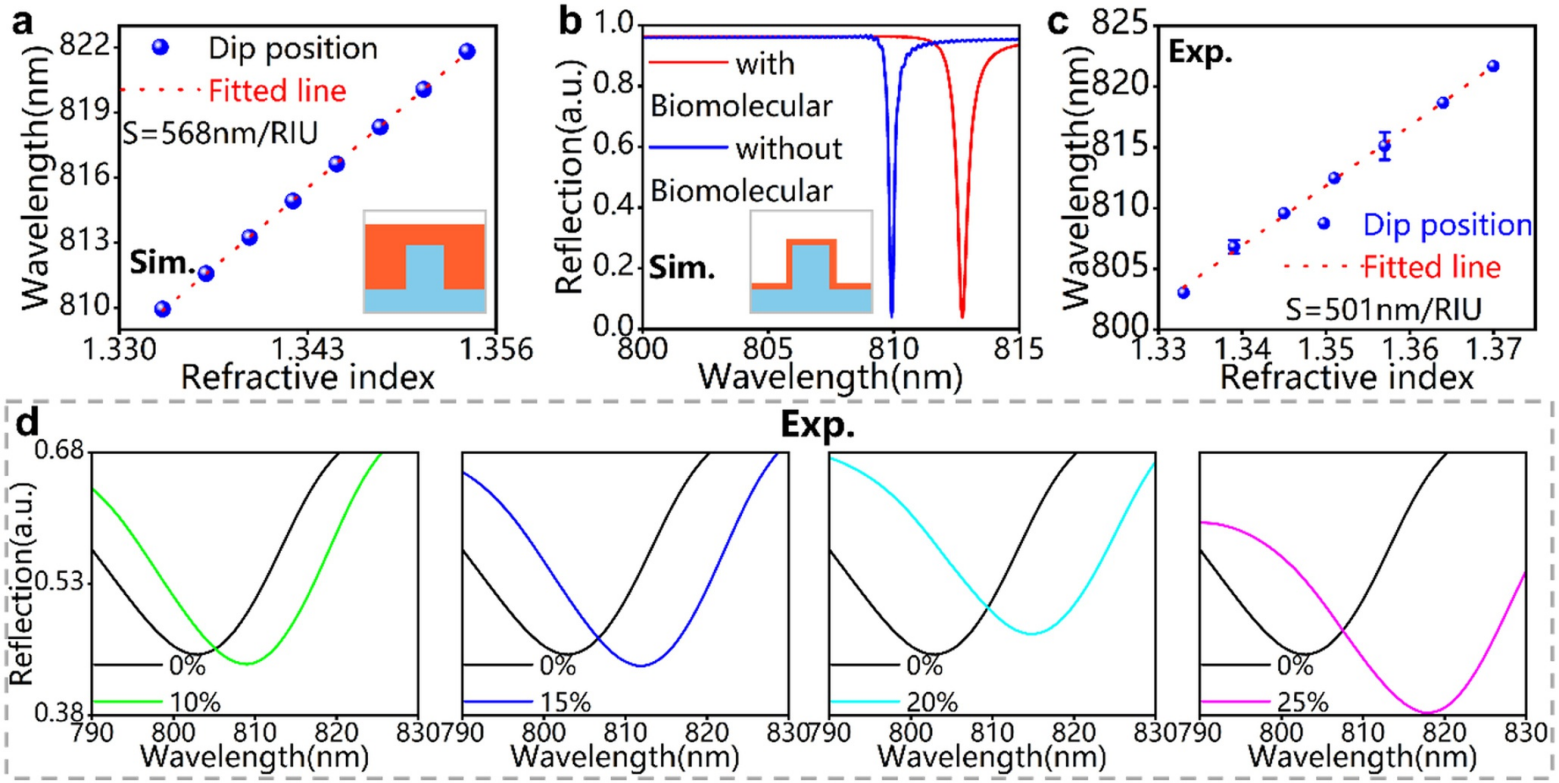
Refractive index sensing characteristics for the NTA. (a) Simulated resonance wavelength of the D_1_ mode varies with the surrounding refractive index (RI) from 1.333 to 1.356. The red‐dashed curve represents a linear fit with a bulk sensitivity of 568 nm/RIU. (b) Simulated resonance D_1_ dip as a function of the thickness of the surface biomolecular layer, reflecting the surface sensitivity of the NTA. (c) Measured resonance D_1_ dip as a function of the RI from the different aqueous glycerol solutions, and the experimental bulk RI sensitivity of NTA was evaluated to be 501 nm/RIU. (d) Measured spectra of the NTA in glycerol solutions with different concentrations (0%, 10%, 15%, 20%, and 25%).

Experimentally, a microarea spectral measurement setup based on an integrated microscope and tungsten lamp illumination is built (Supporting Information S1: Figure S9b), and the sensor is immersed in glycerol aqueous solutions with mass fractions of 0%, 5%, 10%, 15%, 20%, and 30%, corresponding to refractive indices from 1.333 to 1.384. Before measurements with glycerol solutions of varying concentrations, the NTA was cleaned using the following protocol: the sample surface was rinsed three times with deionized (DI) water (10 min each) to remove any residual high‐concentration solution completely, then gently blown dry with dust‐free nitrogen gas. Afterward, the reflection spectrum of DI water (0% glycerol) was recorded, and measurements for the next concentration began only after confirming baseline recovery.

As the surrounding refractive index increases, the D_1_ mode exhibits a clear redshift with good linearity, and linear fitting yields an experimental refractive‐index sensitivity of 501 nm/RIU. The linear correlation coefficient (*R* ^2^) reaches 0.998. In addition, the experimental results indicate an FWHM of 12 nm, yielding an experimentally measured FOM of 42 RIU^−1^ and a *Q* of 70. As summarized in Table S1, our NTA exhibits competitive experimental sensitivity and FOM relative to state‐of‐the‐art metasurface sensors. Although some theoretical designs promise higher simulated FOM, they often lack experimental verification or require low‐throughput fabrication. By contrast, our sensor integrates competitive sensing performance, polarization‐stable response, and scalable nanoimprint manufacturability, making it attractive for practical sensing applications.

The experimental data thus validate the core sensing capability predicted by simulation and confirm that the resonance remains sufficiently sharp and stable under liquid‐phase measurement conditions. To demonstrate label‐free biosensing capability, the NTA sensor is further tested with aqueous solutions of creatinine and hemoglobin. Figure 5a shows a schematic of an NTA‐based biomolecule sensing platform for spectral analysis. In creatinine solutions with increasing concentration, the reflection spectra of D_1_ mode show a significant redshift relative to the deionized water baseline (Figure 5b,c, Supporting Information S1: Figure S12a), and the resonance wavelength shifts approximately linearly with concentration, yielding a sensitivity of 127.5 nm·(μg/L)^−1^ (Figure 5d). We also measured the reflection spectra of the NTA in hemoglobin solutions with different concentrations (Figure 5e–g). Some spectra with other concentrations are also plotted in Supporting Information S1: Figure S12b. It was observed that hemoglobin solutions exhibit a larger redshift than creatinine, attributed to their higher refractive index, which results in a stronger optical perturbation of the surface‐localized field. The variation in refractive index with concentration is reported in Ref. [51]. Linear fitting of the hemoglobin measurements yields a sensitivity of 354.7 nm·(μg/L)^−1^ (Figure 5h). It should be noted that NTA's response to creatinine and hemoglobin solutions is based on changes in the bulk refractive index, a label‐free detection method without molecular recognition. Collectively, the biosensing results confirm that toroidal‐dominant D1‐mode resonance provides a sensitive optical readout for biomolecular concentration variations in aqueous environments. The NTA shows a sensitive response to concentration changes as small as 5 × 10‐3 μg/L in both creatinine and hemoglobin solutions, indicating a limit of detection (LOD) below this value that meets the requirements for practical clinical diagnostics [52]. Although practical sensitivity can be influenced by external factors, such as spectral resolution, temperature fluctuations, and mixture uniformity, the NIL‐fabricated platform offers a promising path toward low‐cost scalable nanophotonic biosensors for applications including medical diagnostics and environmental monitoring.

**FIGURE 5:**
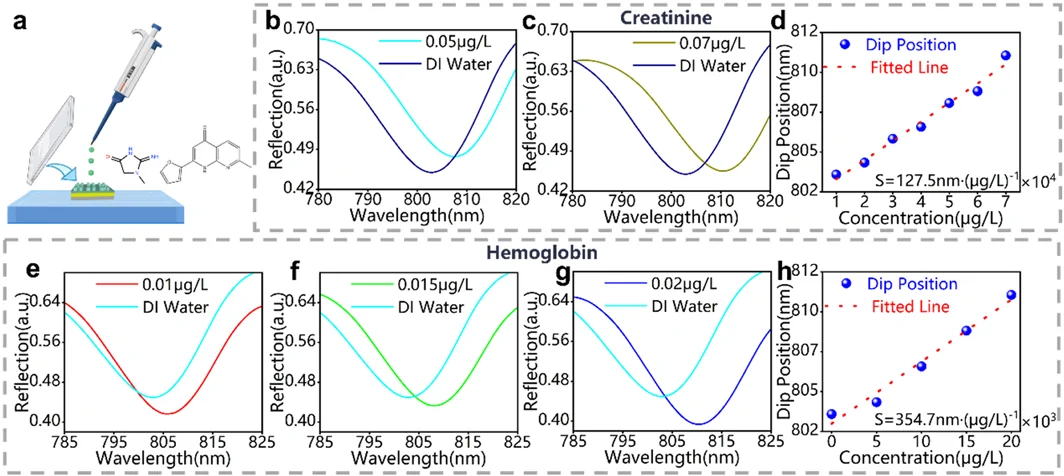
Label‐free biosensing characteristics of the NTA. (a) Schematic of the NTA‐based biomolecule sensing platform for spectral analysis. The reflection spectra of the D_1_ mode within the creatinine solutions of (b) 0.05 and (c) 0.07 μg/L, respectively. (d) The resonant wavelength shift as a function of creatinine concentration, with a linear fit yielding a sensitivity of 127.5 nm·(μg/L)^−1^. (e–g) Measured reflection spectra of the NTA in hemoglobin solutions with concentrations of 0.01, 0.015, and 0.02 μg/L, respectively. (h) Resonant wavelength shift as a function of hemoglobin concentration, with a linear fit yielding a sensitivity of 354.7 nm·(μg/L)^−1^.

## 3 Conclusion

We have proposed and experimentally demonstrated a NTA‐based biosensing platform via a two‐step nanoimprint lithography process. The device supports two resonance modes, among which the D_1_ mode at 810 nm exhibits a pronounced Fano profile with a high *Q* = 3124 and a FWHM of 0.26 nm. The experimentally measured resonance of the fabricated device exhibits a FWHM of approximately 12 nm, corresponding to a *Q* factor of about 70. Further multipole decomposition confirms that the D_1_ mode is dominated by a TD, with conventional ED and MD strongly suppressed, indicating that the high‐Q resonance arises from multipole‐engineered radiation cancellation. The field distributions show strong enhancement at the nanopillar surface and in the gap regions, maximizing analyte‐field overlap and directly enabling high sensitivity. The NTA experimentally demonstrates a 501 nm/RIU sensitivity with a FOM of 42 RIU^−1^ in glycerol solutions. Moreover, as a label‐free biosensing platform, the NTA achieves sensitivities of 127.5 nm·(μg/L)^−1^ for creatinine and 354.7 nm·(μg/L)^−1^ for hemoglobin. Notably, the NTA design provides polarization‐insensitive performance over 0°–90°, enhancing operational stability and eliminating the need for polarization alignment in practice. This work demonstrates the practical potential of toroidal‐dipole‐assisted high‐Q NTA for next‐generation biosensing and identifies further opportunities to integrate toroidal multipolar engineering with manufacturable nanophotonic platforms.

## Author Contributions

**Hongsen Zhao:** writing – original draft, writing – review and editing, methodology, conceptualization, investigation, data curation, software, validation, formal analysis, supervision. **Shuai Han:** data curation, methodology, conceptualization, investigation, writing – original draft, writing – review and editing, formal analysis, software. **Youpeng Zhuo:** investigation, validation, software, data curation, formal analysis, methodology, conceptualization. **Ang Xu:** formal analysis, investigation, validation. **Zhixin Wan:** validation, methodology, formal analysis. **Yimin Chen:** validation, methodology, formal analysis. **Peiqing Zhang:** validation, methodology, formal analysis. **Qiushun Zou:** formal analysis, data curation, supervision, resources, project administration, writing – review and editing, funding acquisition, validation, visualization, investigation. **Xiang Shen:** resources, project administration, formal analysis, visualization.

## Conflicts of Interest

The authors declare no conflicts of interest.

## Supporting information


Supporting Information S1

## Data Availability

The data that support the findings of this study are available from the corresponding author upon reasonable request.
