# Supplementary material for: Toroidal Dipole‐Dominated High‐Performance Optical Sensors via Nanoimprinted Tetramer Architectures
Source: Nanophotonics. 2026 Oct 21;15(20):e70336. doi: 10.1002/nap2.70336 (PMC13686256; doi:10.1002/nap2.70336)
Supplement: Supplementary file 1 [file NAP2-15-e70336-s001.docx]

Supporting Information

**Toroidal dipole-dominated high-performance optical sensors via nanoimprinted tetramer architectures**

Hongsen Zhao ^1,2,#^ | Shuai Han^1,2,#^ | Youpeng Zhuo^1,2^ | Ang Xu ^1,2^ | Zhixin Wan^3^ | Yimin Chen^1,2^ | Peiqing Zhang ^2^ | Qiushun Zou,^1, 2,^ * | Xiang Shen ^1, 2^

^1^Faculty of Electrical Engineering and Computer Science, Ningbo University, Ningbo 315211, China

^2^Zhejiang Key Laboratory of Advanced Optical Functional Materials and Devices, Engineering Research Center for Advanced Infrared Photoelectric Materials and Devices of Zhejiang Province, Ningbo University, Ningbo 315211, China

^3^Key Laboratory of Green Fabrication and Surface Technology of Advanced Metal Materials, Ministry of Education, School of Materials Science and Engineering, Anhui University of Technology, Ma'anshan 243002, China

^#^These authors contributed equally to this work

* [zouqiushun@nbu.edu.cn](mailto:zouqiushun@nbu.edu.cn)

**Cartesian multipole decomposition:**

To elucidate the physical origin of the resonances, a Cartesian multipole decomposition is performed by calculating far-field scattering contributions from the ED, MD, toroidal dipole (TD), electric quadrupole (EQ), and magnetic quadrupole (MQ) components. These multipole decomposes the far-field radiated power, which can be expressed as

$$\begin{aligned} I_{ED}=\frac{2\omega^{4}}{3c^{3}}\left| \frac{1}{i\omega}\int\vec{J}ⅆ^{3}r \right|^{2}\#\left( 1 \right) \end{aligned}$$

$$\begin{aligned} I_{MD}=\frac{2\omega^{4}}{3c^{3}}\left| \frac{1}{2c}\int\left( \vec{r}\times\vec{d} \right)ⅆ^{3}r \right|^{2}\#\left( 2 \right) \end{aligned}$$

$$\begin{aligned} I_{TD}=\frac{2\omega^{6}}{3c^{5}}\left| \frac{1}{10c}\int\left[ \left( \vec{r}\cdot\vec{J} \right)\vec{r}-2r^{2}\vec{J} \right]ⅆ^{3}r \right|^{2}\#\left( 3 \right) \end{aligned}$$

$$\begin{aligned} I_{EQ}=\frac{\omega^{6}}{5c^{5}}\sum\left| \frac{1}{i2\omega}\int\left[ r_{\alpha}J_{\beta}+{r_{\beta}J}_{\alpha}-\frac{2}{3}\left( \vec{r}\cdot\vec{J} \right)\delta_{\alpha\beta} \right]ⅆ^{3}r \right|^{2}\#\left( 4 \right) \end{aligned}$$

$$\begin{aligned} I_{MQ}=\frac{\omega^{6}}{40c^{5}}\sum\left| \frac{1}{3c}\int\left[ \left( \vec{r}\times\vec{J} \right)_{\alpha}r_{\beta}+\left( \vec{r}\times\vec{J} \right)_{\beta}r_{\alpha} \right]ⅆ^{3}r \right|^{2}\#\left( 5 \right) \end{aligned}$$

Where *c* is the speed of light, *ω* is the angular frequency, $\vec{J}$ is the induced volume current density, $\vec{r}$ is the position vector, and $\alpha,\beta=x,y$. The scattered powers of ED, MD, TD, EQ, and MQ as a function of the wavelength are shown in Fig. 1(d).

**Fig. S1.** Fano fitting of the D_2_ mode (red dashed line), consistent with the simulated spectrum (blue solid line).

**Fig. S2.** Cross-sectional electric-field vector distributions of the D1 mode. **(a)** Electric-field distribution in the *x*-*z* plane at *y*=300 nm. **(b)** Electric-field distribution in the *x*-*z* plane at *y*=-300 nm. The enlarged views reveal pronounced local circulating electric-field patterns within the individual nanopillars, consistent with the toroidal-dipole-dominated nature of the D_1_ mode.

**Fig. S3.** Optical properties of D_2_ mode. (a) Cartesian multipole decomposition of D_2_ mode. (b)The components of the electric quadrupole and magnetic quadrupole in different directions.

**Fig. S4.** Multipole decomposition of the tetramer structure without the gold mirror, silicon substrate, and PS waveguide.

**Fig. S5.** (a-b) The total electric field distribution and normalized electric field intensity map for D_2_ mode. (c-d) The electric field distribution of the D_2_ mode at the cross-sections of z=300 nm, x=300 nm, and y=300 nm. e. Electric-field vector distributions of the D_2_ mode in two representative *x*-*z* cross sections. Thin arrows indicate the in plane electric-field directions, while thick circular arrows illustrate the local electric-field circulation within the nanopillars.

**Fig. S6.** The full dispersion curves of NTA for varying *l* and *r*.

**Fig. S7.** Optical properties of D_3_ mode. (a)The simulated spectrum of NTA at *h*=400nm. (b) Cartesian multipole decomposition of D_3_ mode. (c-d) Electric field distributions of D_3_ mode with *h*=400nm. (e). Electromagnetic field distributions of the D_3_ mode. Top: electric-field magnitude and in-plane electric-field vectors in the *y*-*z* plane cross section at (x=-300) nm. Bottom: magnetic-field magnitude and in-plane magnetic-field vectors in the *x*-*z* plane cross section at (y=300) nm. Colors represent the respective field magnitudes. Black and red vector arrows indicate the in-plane electric- and magnetic-field directions, respectively. Thick black curved arrows schematically indicate local electric-field circulation.

Fig. S8. Optimal geometric parameters of the NTA. (a) The reflection spectra of the NTA with *x* varying from 100 to 300 nm. (b)The FWHM and Q of the D_1_ mode with *x*. (c)The reflection spectra of the NTA with *y* from 100 to 300 nm. (d)The FWHM and Q of the D_1_ mode changes as functions of *y*. (e)The reflection spectra of the NTA with *p* from 1150 to 1250 nm. (f)The FWHM and Q of the D_1_ mode as functions of *p*.

**Fig. S9.** Microscopic morphologies and reflection spectral measurement system. (a) The CCD images of NTA. (b) Schematic diagram of the reflection spectral measurement system. Light from a tungsten source was linearly polarized by a polarizer and directed onto the sample through a lens and a 50× objective with a nominal NA of 0.55. The optical axis of the objective was aligned along the surface normal of the sample, while the actual illumination had a finite angular distribution determined by the filling of the objective pupil. The effective illumination NA was not independently calibrated. The same 50× objective was used to collect the reflected light, which was subsequently coupled into an Ocean fiber spectrometer with a spectral resolution of 0.76 nm.

**Fig. S10.** To investigate the influence of gold material losses on the resonance characteristics, the permittivity of gold was adjusted to ***Re(ε)* & *Im(ε)***, ***0.9Re(ε)* & *2Im(ε)***, and ***0.8Re(ε)* & *3Im(ε)***, respectively. The corresponding resonance spectra were then obtained, and the **Q factor** and **full width at half maximum (FWHM)** of the D_1_ resonance.

**Fig. S11.** The components of the toroidal dipole in different directions.

**Fig. S12.** Biosensing sensitivity characterization of the NTA. (a) Spectra of NTA in creatinine solutions of different concentrations. (b) Spectra of NTA in hemoglobin solutions of different concentrations.

**Table S1.** Comparison of the reported refractive-index sensing performance of representative metasurface sensors based on experimental and numerical studies.

| **Nanostructures** | **Resonance mechanism** | **Sensitivity (nm/RIU) Exp.** | **Sensitivity (nm/RIU) Sim.** | **FOM(RIU⁻¹) Exp.** | **FOM(RIU⁻¹) Sim.** | **Waveband (nm)** | **Ref.** |
| --- | --- | --- | --- | --- | --- | --- | --- |
| Au nanohole array on a hybrid substrate | Plasmonic EOT resonance | 671 | / | ≈42 | / | 900 | [1] |
| Silicon high-contrast grating | Guided-mode resonance | 409 | / | 293 | / | NIR | [2] |
| Asymmetric rectangular a-Si pillar array | MTD-mediated quasi-BIC | 413 | / | 66 | / | 1541 | [3] |
| Triangular-hole silicon nanodisk array | Fano quasi-BIC | / | 248 | / | 3815 | / | [4] |
| Si-SiO₂-Al hybrid nanoantenna array | Hybrid metal-dielectric multipolar resonance | 208 | / | / | / | NIR | [5] |
| Silicon crescent metasurface | Fundamental and higher-order quasi-BIC | 326 | / | 1.2 | / | VIS | [6] |
| Displaced dual-lattice silicon nanodisk array | Nonlocal quasi-BIC | 407 | / | 296 | / | NIR | [7] |
| Chiral dielectric metasurface | TD-mediated chiral quasi-BIC | / | 235 | ^/^ | 20900 | 1420 | [8] |
| Metal-dielectric yurt tetramer | SPR, d-qBIC and m-qBIC | / | 500.94 | / | 491.12 | 808 | [9] |
| Nanoimprinted tetramer architecture | Toroidal-character Fano resonance | 501 | 568 | 42 | 2192 | 810 | This work |

**Cite this work**

1. A. E. Cetin, D. Etezadi, B. C. Galarreta, et al., "Plasmonic nanohole arrays on a robust hybrid substrate for highly sensitive label-free biosensing," Acs Photonics 2(8), 1167–1174 (2015).

2. L. Y. Beliaev, S. Kim, B. F. S. Nielsen, et al., "Optical biosensors based on nanostructured silicon high-contrast gratings for myoglobin detection," ACS Appl. Nano Mater. 6(13), 12364–12371 (2023).

3. N. Li, H. Chen, Y. X. Zhao, et al., "Ultrasensitive metasurface sensor based on quasi-bound states in the continuum," Nanophotonics 14(4), 485–494 (2025).

4. C. Zhao, Y. P. Huo, T. Liu, et al., "All-dielectric metasurface with multiple Fano resonances supporting high-performance refractive index sensing," J Opt Soc Am B 41(1), 36–45 (2024).

5. D. Ray, T. Raziman, C. Santschi, et al., "Hybrid metal-dielectric metasurfaces for refractive index sensing," Nano Lett. 20(12), 8752–8759 (2020).

6. J. Wang, J. Kühne, T. Karamanos, et al., "All-dielectric crescent metasurface sensor driven by bound states in the continuum," Adv. Funct. Mater. 31(46), 2104652 (2021).

7. G. Y. Li, and Y. H. Liu, "Homogeneous and significant near-field enhancement in all-dielectric metasurfaces for sensing applications," Adv. Opt. Mater. 12(22), 2400425 (2024).

8. Z. L. Hu, W. Su, J. L. Shi, et al., "High-Q chiral metasurface with quasi-BIC for tunable circular dichroism and refractive index sensing," J. Mater. Chem. C 14(9), 3540–3546 (2026)

9. S. Lv, P. Tuersun, S. Li, et al., “Refractive Index Sensing Properties of Metal–Dielectric Yurt Tetramer Metasurface,” Nanomaterials 15 (20), 1570 (2025).
